# Pro-repair properties of a human embryonic stem cell-derived astrocyte cell therapy in demyelinating disorders

**DOI:** 10.1016/j.stemcr.2026.102960

**Published:** 2026-06-11

**Authors:** Lihi Sofer Stepanov, Nina Fainstein, Marva Lachish, Tal Ganz, Yoel Shor, Gal Lebiush Shalom, Shir Klein-Lavi, Michal Izrael, Debora Steiner, Benjamin E. Reubinoff, Michel Revel, Tamir Ben-Hur

**Affiliations:** 1 Faculty of Medicine, Hebrew University of Jerusalem, Jerusalem, Israel; 2 The Department of Neurology, The Agnes-Ginges Center for Human Neurogenetics, Hadassah-Hebrew University Medical Center, Jerusalem, Israel; 3 Neurodegenerative Diseases Department, NewcelX Ltd, The Circle 6 | P.O. Box, 8058 Zurich, Switzerland; 4 Hadassah Human Embryonic Stem Cells Research Center, The Goldyne-Savad Institute of Gene Therapy, Hadassah-Hebrew University Medical Center, Jerusalem, Israel

**Keywords:** human embryonic stem cells, astrocytes, demyelinating diseases, CNS repair, oligodendrocyte progenitor cells, microglia, cell therapy, trophic properties

## Abstract

Remyelination failure in demyelinating diseases is driven by inefficient myelin debris clearance, impaired oligodendrocyte progenitor cell (OPC) differentiation into mature oligodendrocytes, and the absence of a sustained pro-repair inflammatory environment. Effective remyelination requires a pro-repair inflammatory environment that supports myelin debris removal and promotes OPC maturation. We examined whether AstroRx, a clinical-grade human embryonic stem cell (hESC)-derived astrocyte therapy, addresses these barriers. In co-cultures *in-vitro*, AstroRx enhanced microglial phagocytosis of myelin debris, extended lymphocyte proliferation, and survival without excessive activation, and enhanced murine and hESC-derived OPC differentiation. In a lysolecithin-induced demyelination model *in-vivo*, intraventricular-delivered AstroRx promoted microglial-mediated myelin debris clearance and oligodendrogenesis in the peri-lesion white matter. These findings demonstrate that AstroRx sustains a pro-repair effect via a bystander mechanism, maintaining an inflammatory milieu, facilitating myelin debris removal, and oligodendrogenesis. With demonstrated clinical safety, scalability, and multi-targeted reparative effects, AstroRx offers a promising off-the-shelf cell therapy for chronic demyelination.

## Highlights

• Human astrocytes (AstroRx) promote phagocytosis of myelin debris by microglial cells
• AstroRx enhances lymphocyte survival and proliferation without excessive activation
• AstroRx drives OPC differentiation and accelerates oligodendrocyte maturation
• AstroRx may serve as a pro-remyelination therapy in demyelinating disorders

## Teaser

Corresponding author and colleagues show that a clinical-grade hESC-derived astrocyte cell therapy (AstroRx) enhances myelin debris clearance by microglia, supports lymphocyte-mediated immune regulation, and accelerates OPC differentiation into oligodendrocytes in co-cultures *in-vitro* and in a demyelination model *in-vivo*. Acting through a bystander mechanism, AstroRx addresses key barriers to repair in chronic demyelination, highlighting its translational promise for demyelinating disorders.

## Introduction

Demyelinating diseases, such as multiple sclerosis (MS), are characterized by immune-mediated chronic demyelination, leading to irreversible axonal degeneration and neurological disability (Franklin and Ffrench-Constant, 2008). While remyelination is a natural reparative process, it is often inefficient in these diseases, contributing to progressive decline and long-term disability (Lassmann, 2019). Despite the availability of systemically acting disease-modifying therapies, there remains an unmet need for regenerative approaches, as current treatments do not directly promote myelin regeneration or functional recovery (Bourdette and Wooliscroft, 2024). Remyelination failure is largely attributed to (1) the absence of a pro-reparative inflammatory response, which impairs microglial function and oligodendrocyte progenitor cell (OPC) recruitment (Miron et al., 2013); (2) inefficient removal of myelin debris, creating a non-permissive environment for remyelination; and (3) failure of OPC to differentiate into myelinating oligodendrocytes, resulting in chronic demyelination and axonal loss (Kotter et al., 2006; Franklin and Simons, 2022). Overcoming these barriers is essential to developing effective regenerative therapies for chronic demyelinating diseases.

Stem cell-based therapies have been highlighted for their immunomodulatory and trophic properties, reducing harmful neuroinflammation and contributing to tissue repair (Ben-Hur, 2011; Smith et al., 2021). Both murine and human neuro-glial precursor cells exert immunomodulatory and neuroprotective effects in demyelinating diseases (Aharonowiz et al., 2008; Nishri et al., 2020). Mesenchymal stem cells (MSC), for example, modulate immune responses by shifting macrophages and microglia toward a pro-repair phenotype, reducing excessive inflammation, and secreting neurotrophic factors, thereby supporting neuronal survival (Uccelli et al., 2013). However, MSC production for autologous transplantation is laborious and expensive, with low scalability potential (Galipeau and Sensébé, 2018).

A cell therapy addressing remyelination failure should satisfy two criteria: (1) produced as an “off-the-shelf” product, with proper scalability and standardization, ensuring consistent clinical application and large-scale accessibility; (2) target the biological mechanisms underlying remyelination failure.

Astrocytes are key regulators of central nervous system (CNS) homeostasis, exerting neuroprotective, immunomodulatory, and trophic effects (Lassmann and Bradl, 2017). They are candidates for cell therapy in demyelinating diseases due to their ability to create a supportive microenvironment that facilitates oligodendrocyte maturation (Nutma et al., 2020; Molina-Gonzalez et al., 2023). By influencing microglial activation and secreting trophic factors, astrocytes help regulate oligodendrogenesis, positioning them as neuroregeneration mediators (Lloyd and Miron, 2019). AstroRx is a clinical-grade astrocyte cell therapy derived from human embryonic stem cells (hESC), offering a scalable, “off-the-shelf” therapeutic approach that may target multiple pathological barriers in chronic demyelinating diseases. AstroRx has been shown to clear excessive glutamate, reduce oxidative stress, secrete various neuroprotective factors, and act as an immunomodulator, reinforcing its potential to modulate neuroinflammatory and neurodegenerative processes (Izrael et al., 2018). Notably, in the Phase I/IIa clinical trial for amyotrophic lateral sclerosis (ALS), AstroRx demonstrated a high safety profile and potential therapeutic efficacy (Gotkine et al., 2023).

In this study, we investigated AstroRx ability to overcome key biological barriers limiting remyelination. We show *in-vitro* and *in-vivo* that AstroRx accelerates myelin debris removal, maintains an active inflammatory state, and promotes both murine OPC and hESC-OPC differentiation into mature oligodendrocytes. We suggest that AstroRx may promote a pro-repair inflammatory environment, with potential as a novel therapeutic approach for chronic demyelinating diseases.

## Results

### AstroRx promote microglia phagocytotic activity *in-vitro*

**Figure 1:**
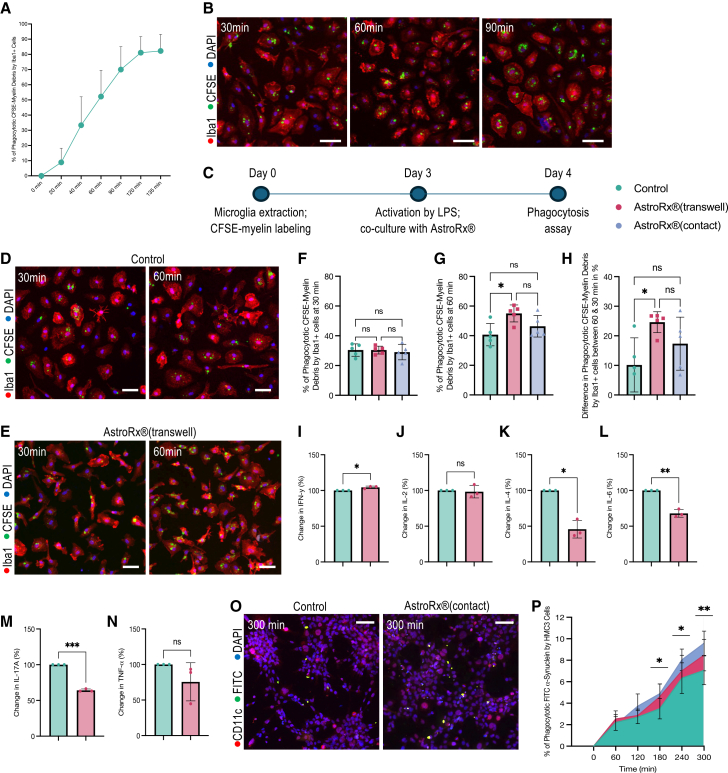
AstroRx promote microglia phagocytotic activity *in-vitro* (A) Microglial phagocytosis calibration assay: LPS-activated microglia were fed CFSE-labeled myelin debris at 20, 40, 60, 90, 120, and 135-min time points; *n* = 3 experiments (exp). (B) Calibration experiment of microglial phagocytic activity by CFSE-labeled myelin debris engulfment into Iba1^+^ cells. (C) Time-course of microglia phagocytic activity experiment. (D and E) Immunofluorescence analysis of microglial phagocytic activity by CFSE-labeled myelin debris engulfment into Iba1^+^ cells at 30 and 60 min in (D) control and (E) AstroRx(transwell) conditions. (F) AstroRx did not affect microglial phagocytotic activity in the first 30 min; *n* = 5 exp, *p* = 0.861. (G) AstroRx promoted microglial phagocytosis at the 60 min time point; *n* = 5 exp. (H) AstroRx sped up myelin debris uptake between 60 and 30 min; *n* = 5 exp. (I–N) CBA analysis of AstroRx effect on microglial inflammatory response, (I) AstroRx slightly increased INF-γ secretion; (J) IL-2 secretion was unaffected; (K–M) AstroRx decreased IL-4, IL-6, and IL-17A secretions; (N) TNF-α secretion was unaffected; *n* = 3 exp. (O) Immunofluorescence analysis of CD11c^+^ HMC3 phagocytotic activity of FITC-α-synuclein, in control and AstroRx(contact) conditions at 300 min. (P) AstroRx increased HMC3 phagocytosis in a time-dependent manner, *n* = 3 exp. Scale bars, 50 μm. Significance was determined by one-way ANOVA with Tukey’s post hoc test (F–H), student’s paired *t*test (I–N), and two-way ANOVA (P).

To examine whether AstroRx modulates the pro-reparative inflammatory process, we first examined its effect on microglia phagocytosis of myelin debris. CD11b^+^ microglia were isolated from naive Biozzi ABH mice, activated with lipopolysaccharide (LPS), and fed with CFSE-labeled myelin debris. Time-lapse microscopy assessed phagocytosis dynamics (Figures 1A and 1B), peaking between 30 and 90 minutes (min), nearing plateau at 90 min. Given our previous findings on the partial dependence of neural precursor cells (NPC) on direct cell contact to exert maximal immunomodulatory effects (Einstein et al., 2007), we investigated whether AstroRx required similar conditions or could exert a bystander effect through secreted factors. LPS-activated microglia were co-cultured with AstroRx in direct contact or separated by a transwell membrane, while control microglia were cultured alone. After 24 h of co-culture, phagocytosis of CFSE-labeled myelin debris was quantified at 30, 60, and 90 min (Figures 1C–1E). Analysis at 30 min revealed no significant difference in phagocytic activity among the groups: control (30.41 ± 4.15%), AstroRx(transwell) (30.41 ± 2.64%), AstroRx(contact) (29.02 ± 5.19%; Figure 1F). At 60 min, microglia co-cultured with AstroRx in transwell exhibited significantly greater phagocytosis (55.02 ± 5.75%) than control (40.71 ± 7.52%) and direct contact (46.35 ± 7.27%; *p* = 0.018; Figure 1G). The difference in myelin debris uptake between 60 min and 30 min was most pronounced in AstroRx(transwell) (24.61 ± 3.54%) versus control (10.16 ± 9.15%) and AstroRx(contact) (17.34 ± 8.97%; *p* = 0.036; Figure 1H), indicating that AstroRx enhances microglial phagocytosis primarily by a robust contact-independent, bystander effect.

To further evaluate AstroRx impact on microglial inflammatory state, cytokine secretion of control and AstroRx transwell groups was analyzed using cytometric bead array (CBA), with control levels normalized to 100% (Figures 1I–1N). INF-γ secretion was mildly elevated by the presence of AstroRx (104.5 ± 1.71%; *p* = 0.045), while IL-2 levels remained unchanged (98.36 ± 8.84%; *p* = 0.779). In contrast, IL-4, IL-6 and IL-17A decreased significantly, (IL-4: 45.80 ± 12.41%, *p* = 0.017, IL-6: 68.00 ± 5.46%, *p* = 0.0096; IL-17A: 64.21 ± 1.84%, *p* = 0.0009). TNF-α levels were not majorly affected (75.62 ± 26.83%; *p* = 0.256), and IL-10 secretion was undetectable in all groups. This cytokine profile suggests that AstroRx support microglia phagocytotic activity while modulating cytokine output, placing microglia in a functionally activated nonpolarized state, which is consistent with reparative activation without excessive inflammation, essential for CNS repair (Cherry et al., 2014).

To extend these findings to a human system, we evaluated the effects of AstroRx on the phagocytosis of FITC-labeled α-synuclein by HMC3, a human microglial cell line. AstroRx co-culture progressively enhanced phagocytosis over time, most prominently in direct contact at 300 min (control (7.07 ± 1.34%), AstroRx(transwell) (8.48 ± 1.47%), AstroRx(contact) (9.58 ± 1.13%), *p* = 0.003; Figures 1O–1P). These findings indicate that AstroRx enhances phagocytic capacity in human microglia, supporting their translational relevance in human systems.

### AstroRx promotes lymphocyte proliferation and survival *in-vitro*

**Figure 2:**
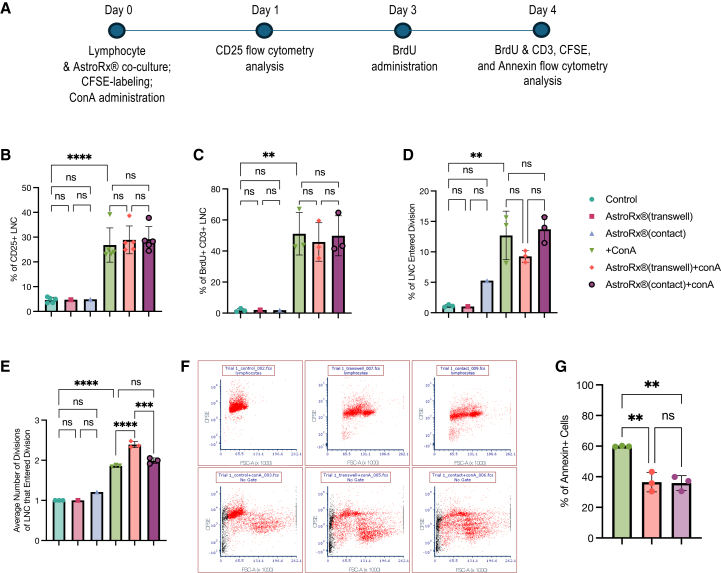
AstroRx promotes lymphocytes proliferation and survival *in-vitro* (A) LNC Experimental Timeline. (B) AstroRx did not alter CD25 expression in unstimulated or ConA-stimulated LNC; *n* = 5 exp, *p* < 0.0001. (C) AstroRx did not affect BrdU incorporation into CD3^+^ LNC in unstimulated and ConA-stimulated LNC; *n* = 3 exp, *p* = 0.002. (D) AstroRx did not affect the fraction of LNC that entered the cell cycle by CFSE analysis; *n* = 3 exp, *p* = 0.0006. (E) AstroRx increased the average number of divisions in ConA-stimulated LNC; *n* = 3 exp. (F) Flow cytometry plots of CFSE-labeled LNC upon treatment with AstroRx(transwell), AstroRx(contact), +ConA, AstroRx(transwell)+ConA, AstroRx(contact)+ConA, and control. (G) AstroRx reduced the fraction of Annexin^+^ LNC; *n* = 3 exp, *p* = 0.289. Significance was determined by one-way ANOVA with Tukey’s post hoc test.

A continued active inflammatory environment is also required for successful repair processes. Therefore, we examined whether AstroRx influences lymphocyte activation, proliferation, and survival, which are hallmarks of a balanced pro-repair inflammatory environment. To assess the effects of AstroRx on lymphocyte function, lymph node cells (LNC) were isolated from naive Biozzi ABH mice, stimulated with concanavalin A (ConA), and co-cultured with AstroRx in transwell or direct contact (Figure 2A). Flow cytometry analyzed LNC activation (by CD25 expression), proliferation (by BrdU incorporation into CD3^+^ LNC and by CFSE content assay), and cell survival (by Annexin-V staining). LNC activation, assessed by CD25 expression after 24 h of co-culture, was not affected by the presence of AstroRx (control (26.82 ± 6.91%), AstroRx(transwell) (28.90 ± 5.62%), AstroRx(contact) (29.20 ± 5.05%); Figure 2B). Furthermore, CD25 expression in unstimulated LNC remained similarly low in control and AstroRx conditions (∼4.6–4.9%). These results indicate that AstroRx does not affect LNC activation, suggesting also that it is not inherently immunogenic.

Proliferation was evaluated using BrdU incorporation into CD3^+^ cells and CFSE content. BrdU incorporation in ConA-stimulated cultures was unaffected by AstroRx (control (51.13 ± 13.70%); AstroRx(transwell) (45.82 ± 12.41%); AstroRx(contact) (49.83 ± 12.89%); Figure 2C) and remained low in unstimulated LNC (∼1.0–2.0%). Similarly, the fraction of LNC that entered the cell cycle, measured by CFSE content, did not significantly differ between groups (control (12.70 ± 3.99%), AstroRx(transwell) (9.25 ± 0.954%), AstroRx(contact) (13.71 ± 2.13%); Figure 2D). The percentage of dividing unstimulated LNC remained similarly low in all groups (∼1.0–5.2%). However, AstroRx did significantly increase the average number of divisions in proliferating lymphocytes (ConA-stimulated control (1.86 ± 0.02), AstroRx(transwell) (2.39 ± 0.08), AstroRx(contact) (1.99 ± 0.07), *p* < 0.0001; Figures 2E and 2F). The number of cell divisions in unstimulated LNC remained similarly low (1.0–1.2).

Analysis of Annexin-V^+^ apoptotic LNC at four days of co-culture (Figure 2G), showed significantly lower percentage of Annexin^+^ cells in the presence of AstroRx (ConA-stimulated control (59.88 ± 0.27%), (AstroRx(transwell) (36.50 ± 6.39%), AstroRx(contact) (35.88 ± 4.80%). This suggests that AstroRx enhances lymphocyte survival, possibly by providing trophic support or modulating apoptosis-related signaling pathways.

Overall, these results suggest that AstroRx is not immunogenic and does not activate lymphocytes, but support their expansion once proliferation has initiated and supports their survival, characteristics associated with long-term immune response regulation (Dombrowski et al., 2017). AstroRx may sustain an active immune environment, potentially contributing to a pro-repair inflammatory process necessary for overcoming chronic demyelination.

### AstroRx promotes murine OPC differentiation to oligodendrocytes *in-vitro*

**Figure 3:**
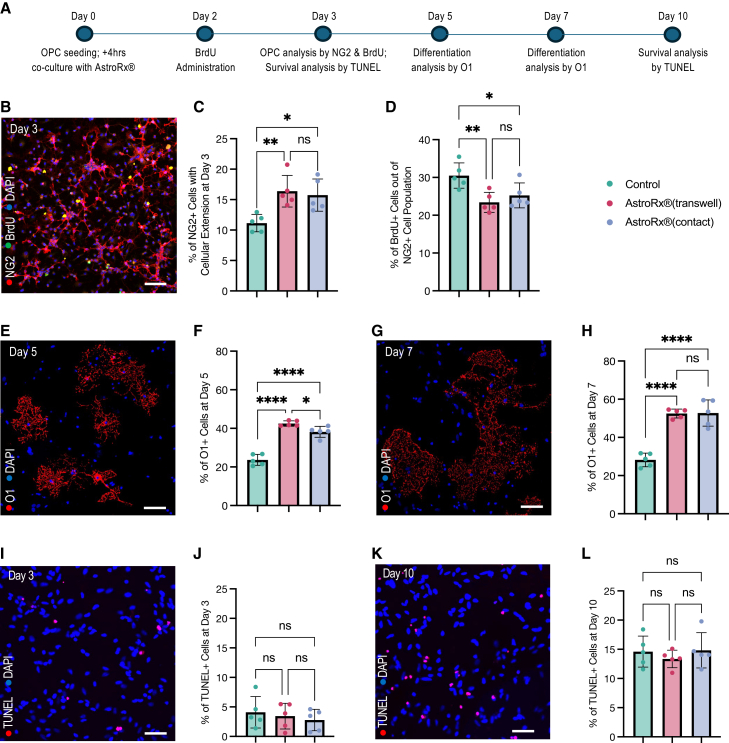
AstroRx promotes murine OPC to differentiate into mature oligodendrocytes *in-vitro* (A) Time course of OPC proliferation, differentiation and survival. (B) Immunofluorescence analysis of OPC differentiation by NG2 and BrdU. (C) AstroRx increased the fraction of the NG2^+^ cell population out of the total cell population. (D) AstroRx decreased BrdU incorporation into NG2^+^ cells. (E and G) Immunofluorescence analysis of OPC differentiation by O1 on days 5 and 7, respectively. (F and H) AstroRx induced OPC differentiation into mature O1^+^ oligodendrocytes on days 5 and 7; Day 5: control (23.67 ± 2.86%), AstroRx(transwell) (42.52 ± 1.41%), AstroRx(contact) (38.19 ± 2.82%). (I and K) Immunofluorescence analysis of OPC survival by TUNEL on days 3 and 10. (J and L) AstroRx did not affect the fraction of apoptotic cells out of the total cell population on days 3 and 10. Scale bars, 50 μm. *n* = 5 exp. Significance was determined by one-way ANOVA with Tukey’s post hoc test.

As the inability of local OPC to respond and differentiate into re-myelinating oligodendrocytes obstructs remyelination (Miron et al., 2013; Kotter et al., 2006), we investigated whether AstroRx directly affects OPC progenitor state, proliferation, differentiation and survival (Figure 3A). Progenitor state and proliferation were assessed by BrdU incorporation into NG2^+^ cells on day 3, oligodendrocyte differentiation was assessed by O1 staining on days 5 and 7 of co-culture, and survival was analyzed on days 3 and 10 using TUNEL staining. NG2^+^ cells constituted the vast majority of DAPI^+^ cells across conditions (control (90.29 ± 8.09%), AstroRx(transwell) (91.10 ± 3.62%), AstroRx(contact) (82.76 ± 4.35%), *p* = 0.222), confirming comparable oligodendroglial enrichment; the slightly elevated NG2^+^ fraction in direct contact (∼80% vs. ∼70% expected from seeding) likely reflects limited OPC proliferation during co-culture. OPC co-cultured with AstroRx exhibited pronounced NG2^+^ cellular extensions, indicating early morphological commitment toward differentiation (control (11.14 ± 1.42%), AstroRx(transwell) (16.38 ± 2.59%), AstroRx(contact) (15.75 ± 2.65%), *p* = 0.007; Figures 3B and 3C). AstroRx decreased NG2^+^ cell proliferation (control (30.50 ± 3.35%), AstroRx(transwell) (23.40 ± 2.64%), AstroRx(contact) (25.24 ± 3.30%), *p* = 0.009; Figure 3D). AstroRx significantly promoted OPC differentiation into mature O1+ oligodendrocytes on days 5 and 7 (*p* < 0.0001 in both analyses, control (28.21 ± 3.56%), AstroRx(transwell) (52.50 ± 2.36%), AstroRx(contact) (52.78 ± 6.87%) on day 7; Figures 3E–3H). In contrast, no significant impact on cell survival by TUNEL staining on day 3 (control (4.09 ± 2.67%), AstroRx(transwell) (3.44 ± 2.18%), AstroRx(contact) (2.78 ± 1.81%), *p* = 0.9245; Figures 3I and 3J) or day 10 (control (14.60 ± 2.66%), AstroRx(transwell) (13.34 ± 1.51%), AstroRx(contact) (14.82 ± 3.02%), *p* = 0.607; Figures 3K–3L). Given the oligodendroglial lineage enrichment of these cultures, TUNEL quantification reflects survival within the OPC lineage. Collectively, these findings suggest that AstroRx enhances OPC differentiation and does not affect cell viability.

To further assess the impact of AstroRx on oligodendrocyte maturation, computerized analysis of fractal dimension (FD) and skeleton was performed to evaluate morphological complexity. NG2^+^ OPC at day 3 and O1^+^ oligodendrocytes at days 5 and 7 were analyzed for the number of endpoints and branch length per cell. On day 3, AstroRx co-culture significantly increased the number of endpoints per NG2^+^ OPC and branch length (Figures S1A and S1B), without altering FD scores (Figures S1C and S1D). By day 5, O1^+^ oligodendrocytes co-cultured with AstroRx had significantly elevated endpoints and branch length (Figures S1E and S1F), along with significantly higher FD scores (Figures S1G and S1H), indicating enhanced morphological complexity. On day 7, endpoint counts showed a modest yet significant increase, while branch length increase was significantly more pronounced (Figures S1I and S1J). However, FD scores were similar across all groups (Figures S1K–S1L). These results suggest that AstroRx accelerates oligodendrocyte maturation, enabling them to reach peak complexity more rapidly. Morphological complexity correlates with enhanced remyelination potential, supporting the hypothesis that AstroRx may contribute to more efficient myelination (Toth et al., 2021).

### AstroRx support repair following lysolecithin-induced demyelination by promoting myelin debris clearance and oligodendrogenesis *in-vivo*

**Figure 4:**
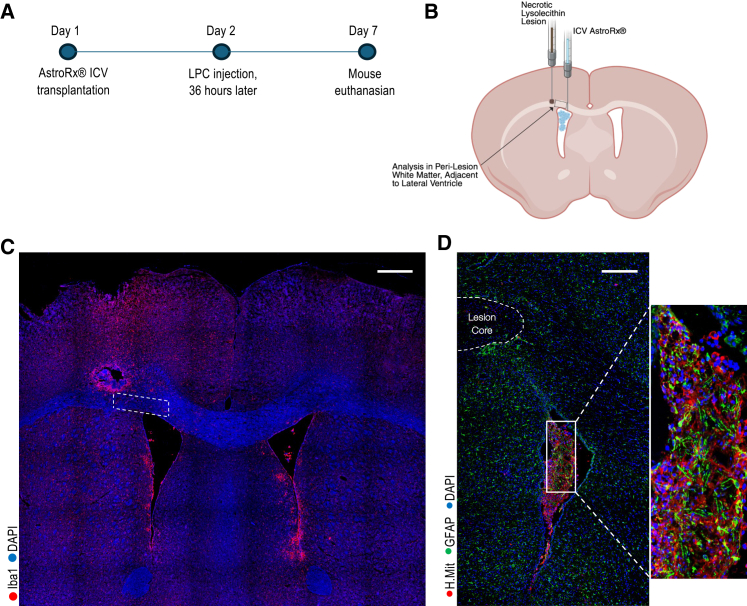
Immunomodulatory and trophic properties of AstroRx in an LPC demyelinating model *in-vivo* (A) Time course of the LPC demyelination. (B) Schematic illustration depicts unilateral AstroRx ICV transplantation and subsequent LPC injection, resulting in a necrotic lesion. The peri-lesion white matter adjacent to the lateral ventricle was analyzed, examining AstroRx and host cell interactions. (C) Immunofluorescence analysis of Iba1^+^ microglia distribution around the necrotic lesion. The dotted region marks the analyzed per-lesion white matter area. (D) Co-immunostaining for GFAP and H.Mit confirmed successful engraftment of AstroRx into the lateral ventricle. Scale bars, 200 μm.

To evaluate the pro-reparative effects of AstroRx in an *in-vivo* demyelination model, we utilized the lysolecithin (LPC)-induced demyelination model, a well-established model of focal demyelination with preserved axonal integrity (Plemel et al., 2018). This model created a focal demyelinated lesion, some distance from transplanted AstroRx, allowing the examination of AstroRx bystander effects on oligodendrogenesis, immune modulation and remyelination potential *in-vivo*. AstroRx was transplanted intracerebroventricularly (ICV) into the lateral ventricle of seven-months-old Biozzi ABH mice. 36 h post-transplantation, LPC was injected into the corpus callosum, 300 μm from the transplantation site, creating a focal necrotic lesion, with an adjacent peri-lesion area of demyelination. On day 7, brains were analyzed within the peri-lesion white matter adjacent to the lateral ventricle. (Figures 4A and 4B). Iba1 staining aided the outline of the peri-lesional area (Figure 4C). To validate AstroRx successful engraftment and survival, co-immunostaining for human mitochondria (H.Mit) and glial fibrillary acidic protein (GFAP) was performed (Figure 4D).

**Figure 5:**
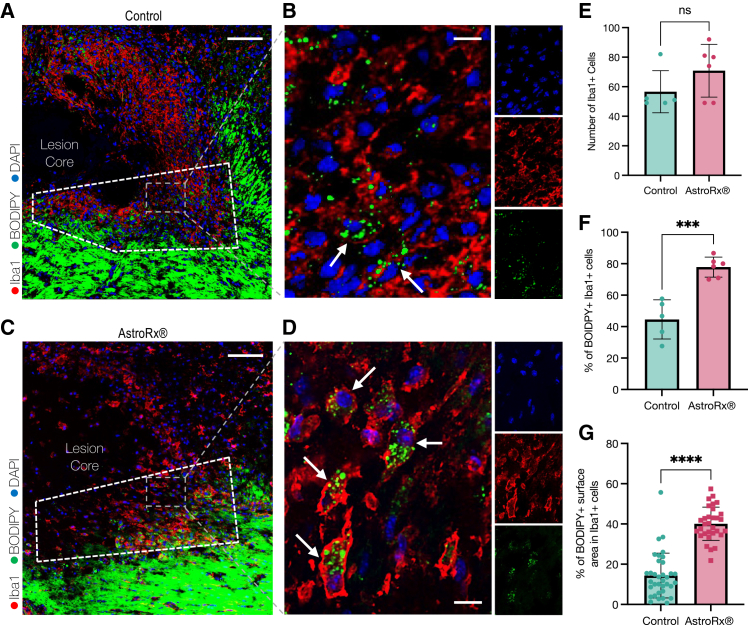
AstroRx promotes microglia phagocytosis to remove myelin debris *in-vivo* (A and C) Low-magnification image of BODIPY^+^ myelin debris and Iba1^+^ cells in control and AstroRx-treated mice, respectively. The peri-lesion demyelinated area is identified by the dotted line (scale bars, 200 μm). (B) High-magnification confocal z-stack of BODIPY^+^ myelin debris near Iba1^+^ cells in control mice. Arrows point at BODIPY remaining largely extracellular (scale bars, 10 μm). (D) High-magnification z-stack with arrows shows internalized BODIPY^+^ myelin debris within Iba1^+^ cells in AstroRx-treated mice (scale bars, 10 μm). (E) AstroRx does not affect the number of Iba1^+^ cells; control (56.60 ± 14.26), AstroRx (70.83 ± 17.88). (F) AstroRx significantly increased the percentage of BODIPY^+^ myelin engulfed by Iba1^+^ cells. (G) AstroRx promotes greater BODIPY^+^ surface area within Iba1^+^ cells; analyzed >150 cells. *n* = 6 mice per condition. Significance was determined by student’s unpaired *t* test.

First, since efficient remyelination requires effective removal of inhibitory myelin debris by microglia (Lloyd and Miron, 2019), we determined whether AstroRx promotes the microglial engulfment of myelin debris *in-vivo*. BODIPY, a lipid droplet-specific marker, was used to identify myelin debris in relation to Iba1^+^ microglia. The strong green staining in Figures 5A and 5C indicate intact myelin in the corpus callosum, whereas in the demyelinated peri-lesion areas, BODIPY particles are found intracellularly in Iba1^+^ microglia of AstroRx transplanted mice, while extracellularly in the control mice (Figures 5A–5D). The total number of Iba1^+^ cells was not significantly altered between the groups (control (56.60 ± 14.26), AstroRx (70.83 ± 17.88), *p* = 0.185; Figure 5E), indicating that AstroRx does not affect microglial recruitment. However, AstroRx significantly increased the fraction of Iba1^+^ cells engulfing BODIPY^+^ myelin debris (control (44.58 ± 12.47%), AstroRx (77.86 ± 6.37%), *p* = 0.0003) and the total BODIPY^+^ surface area within microglia (control (14.39 ± 11.13%), AstroRx (40.07 ± 8.27%), *p* < 0.0001), demonstrating enhanced phagocytotic activity (Figures 5F and 5G). These findings strongly suggest that AstroRx contributes to myelin debris clearance, a critical step for enabling remyelination.

**Figure 6:**
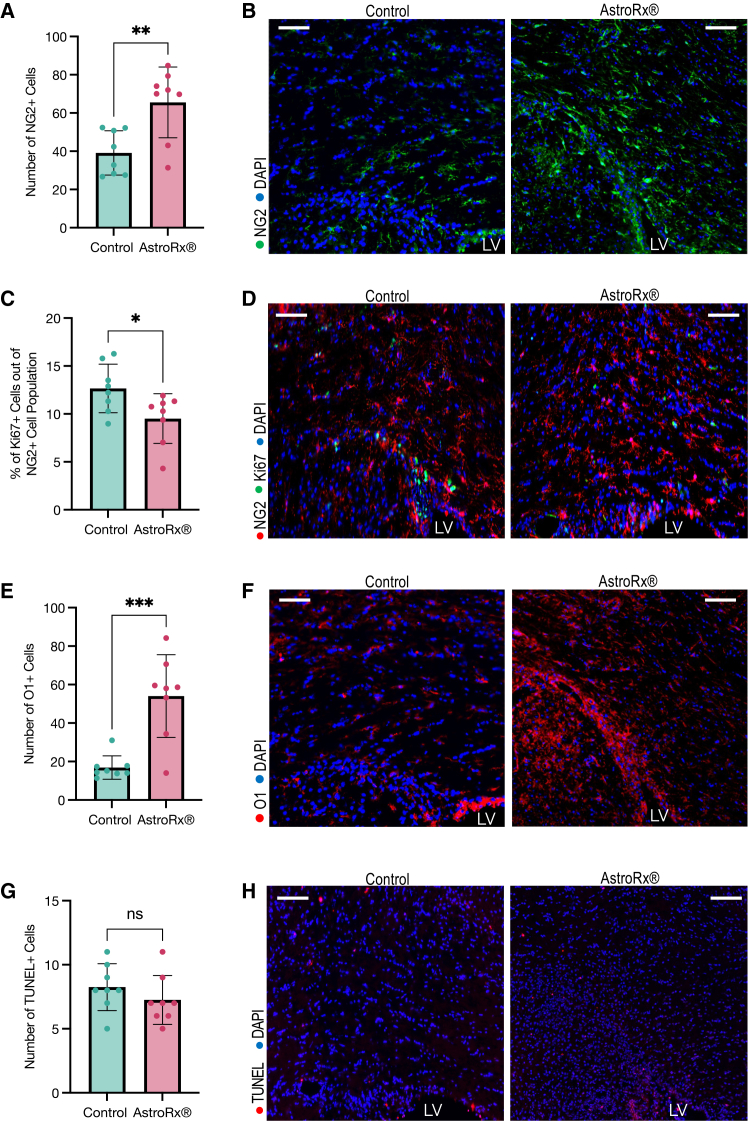
AstroRx increases OPC and oligodendrocytes in lysolecithin-induced demyelinated lesions *in-vivo* (A) AstroRx increases the NG2^+^ cell population in the peri-lesional area. (B) Immunofluorescence analysis of NG2^+^ cells. (C) AstroRx decreases the fraction of Ki67^+^ cells out of the NG2^+^ population. (D) Immunofluorescence analysis of NG2^+^ Ki67^+^ cells. (E) AstroRx promotes the O1^+^ cell population. (F) Immunofluorescence analysis of O1^+^ cells. (G) AstroRx does not affect the TUNEL^+^ cell population. (H) Immunofluorescence analysis of TUNEL^+^ cells. LV indicates the lateral ventricle. Scale bars, 50 μm. *n* = 6 mice per condition. Significance was determined by student’s unpaired *t* test.

To assess AstroRx impact on oligodendrogenesis *in-vivo*, the number of NG2^+^ OPC, Ki67^+^ proliferating OPC, O1^+^ mature oligodendrocytes, and TUNEL^+^ cells were analyzed in the peri-lesional white matter. AstroRx transplantation significantly increased the number of NG2^+^ cells (control (39.11 ± 11.59), AstroRx (65.54 ± 18.49), *p* = 0.004; Figures 6A and 6B), indicating enhanced OPC recruitment. The fraction of Ki67^+^ cells out of the NG2^+^ population was significantly reduced in the AstroRx-treated mice (control (12.66 ± 2.53%), AstroRx (9.51 ± 2.59%), *p* = 0.028; Figures 6C and 6D). AstroRx significantly increased the number of O1^+^ mature oligodendrocytes (control (16.85 ± 6.07), AstroRx (54.05 ± 21.52), *p* = 0.0003; Figures 6E and 6F), confirming amplified OPC differentiation. The total number of TUNEL^+^ cells remained unchanged (control (8.25 ± 1.83), AstroRx (7.25 ± 1.91), *p* = 0.303; Figures 6G and 6H). These findings mirror the *in-vitro* results (Figure 3), reinforcing the hypothesis that AstroRx facilitates OPC recruitment and differentiation, which may contribute to enhanced remyelination potential (Toth et al., 2021).

Lastly, to determine whether enhanced oligodendrocyte differentiation translated into structural repair, myelin internode length was quantified using neurofilament (NF) and BODIPY co-staining (Figure S2). In the adult mouse corpus callosum, internodes range between ∼50 and 150 μm (Young et al., 2013). AstroRx-treated mice exhibited a trend toward shorter myelin internodes compared to control (control (96.78 ± 25.72 μm), AstroRx (85.97 ± 21.98 μm), *p* = 0.0703; Figure S2), consistent with the characteristic shortened internodes observed during remyelination (Franklin and Ffrench-Constant, 2008; Young et al., 2013). Although not statistically significant, this suggests that AstroRx may promote early structural remodeling associated with remyelination.

### AstroRx promotes hESC-OPC differentiation to oligodendrocytes *in-vitro*

**Figure 7:**
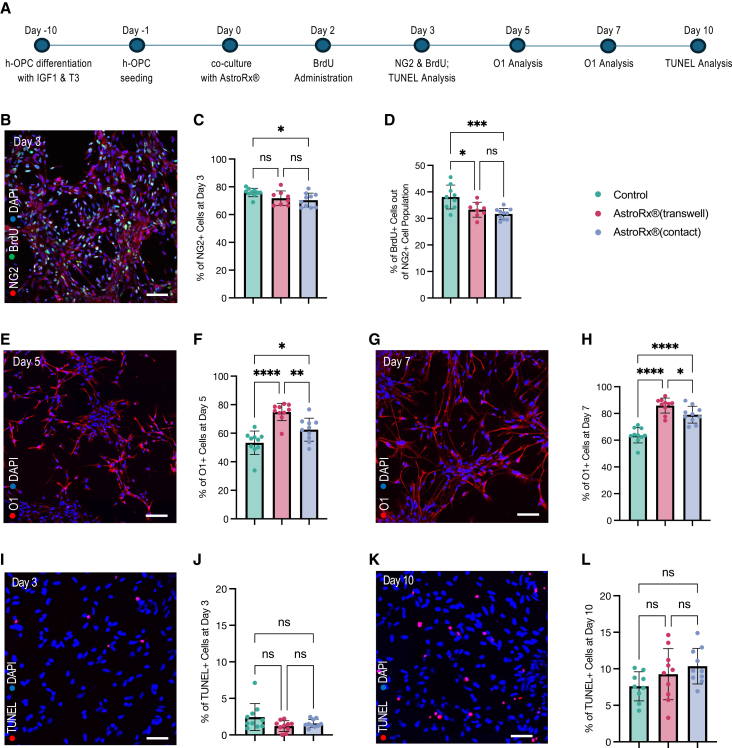
AstroRx promote hESC-OPC to differentiate into mature oligodendrocytes *in-vitro* (A) Time Course of hESC-OPC proliferation, differentiation and survival. (B) Immunofluorescence analysis of OPC differentiation by NG2 and BrdU. (C) AstroRx slightly reduced the fraction of the NG2^+^ cell population out of the total cell population. (D) AstroRx decreased BrdU incorporation into NG2^+^ cells. (E and G) Immunofluorescence analysis of OPC differentiation by O1 on days 5 and 7, respectively. (F and H) AstroRx induced OPC differentiation into mature O1^+^ oligodendrocytes on days 5 and 7. (I and K) Immunofluorescence analysis of OPC survival by TUNEL on days 3 and 10. (J and L) AstroRx did not affect the apoptosis rate on days 3 and 10. Scale bars, 50 μm. *n* = 3 exp. Significance was determined by one-way ANOVA with Tukey’s post hoc test.

To explore the clinical relevance of AstroRx, we examined their effects on oligodendrogenesis in a culture of enriched hESC-OPC, which were further differentiated for 10 days in the presence of IGF1 and T3, and followed the same co-culture experimental timeline explained in Figure 3 (Figure 7A). On day 3, in AstroRx co-cultures, there was a modest reduction in NG2^+^ cell fraction (control (75.92 ± 3.02%), AstroRx®(transwell) (71.79 ± 5.27%), AstroRx(contact) (70.41 ± 5.03%), *p* = 0.031; Figures 7B and 7C). Notably, the cultures were highly enriched in NG2^+^ cells, indicating a predominantly oligodendroglial population and minimal non-lineage contamination. A mild but significant decrease in proliferation rates was noted, by lower BrdU incorporation into NG2^+^ cells (control (38.05 ± 4.49%), AstroRx(transwell) (33.31 ± 2.81%), AstroRx(contact) (31.69 ± 2.04%), *p* = 0.0009; Figure 7D). On days 5 and 7, the percentage of O1^+^ cells were significantly increased in AstroRx co-cultures (day 5: control (53.30 ± 8.19%), AstroRx(transwell) (74.88 ± 6.12%), AstroRx(contact) (62.52 ± 8.05%), *p* < 0.0001; Figures 7E and 7F), and (day 7: control (63.75 ± 5.83%), AstroRx(transwell) (85.89 ± 5.63%), AstroRx(contact) (79.08 ± 6.32%), *p* < 0.0001; Figures 7G and 7H). Lastly, TUNEL staining at days 3 and 10 showed no significant differences in cell survival (day 3: control (2.45 ± 1.84%), AstroRx(transwell) (1.21 ± 0.73%), AstroRx(contact) (1.57 ± 0.55%), *p* = 0.076; Figures 7I and 7J), and (day 10: control (7.60 ± 1.99%), AstroRx(transwell) (9.26 ± 3.50%), AstroRx(contact) (10.36 ± 2.43%), *p* = 0.109; Figures 7K and 7L). These findings suggest that AstroRx promotes the differentiation of hESC-OPC, similarly to murine OPC.

To further characterize the impact of AstroRx on hESC-OPC maturation, fractal and morphological complexity analyses at days 5 and 7 were performed. On day 5, AstroRx significantly increased the number of endpoints per cell, as well as branch length (Figures S3A and S3B). However, FD scores remained similar at this stage (Figures S3C–S3F). By day 7, AstroRx fostered a significant increase in both the number of endpoints and branch length (Figures S3G and S3H), along with significantly elevated FD scores (Figures S3I–S3L), indicating enhanced morphological complexity. Together, these results reinforce the clinical relevance of AstroRx by demonstrating similar pro-differentiation effects in human OPC as observed in murine OPC, further supporting their therapeutic potential in demyelinating conditions.

## Discussion

Demyelinating diseases are characterized by the progressive loss of myelin and impaired repair, both of which contribute to functional decline and neurodegeneration (Franklin and Ffrench-Constant, 2008). While remyelination is a natural reparative process, it is often impaired due to the lack of active inflammation in the chronic stage, persistence of myelin debris, and inability of OPC to differentiate into mature oligodendrocytes (Kotter et al., 2006). The success of remyelination depends on the interplay between immune cells, glial cells, and the extracellular environment, where a controlled pro-repair inflammatory environment is necessary to clear inhibitory myelin debris, modulate immune responses, and promote OPC differentiation (Miron et al., 2013).

Astrocytes are key regulators of CNS homeostasis, regulating microglial function, inflammation, and OPC maturation (Nutma et al., 2020). Therefore, recent research has explored the potential of astrocyte-based cell therapies to promote remyelination (Hastings et al., 2022). Astrocytes support remyelination by promoting OPC differentiation rather than proliferation, as successful remyelination depends on the availability of mature oligodendrocytes (Nutma et al., 2020). In this study, we demonstrate that AstroRx, a hESC-derived astrocyte cell therapy, fosters a pro-repair inflammatory environment, enhances myelin debris clearance, and promotes the differentiation of both murine and human OPC. Together, these findings lay the basis for the clinical translation of AstroRx for promoting repair in demyelinating diseases.

First, the removal of myelin debris is crucial for effective myelin repair, as its accumulation prevents OPC maturation and sustains a pro-inflammatory state, hindering repair. Previous studies showed that enhancing microglial clearance of myelin debris restores OPC regenerative capacity and promotes functional recovery in demyelinated lesions (Kotter et al., 2006; Lloyd and Miron, 2019). We show that AstroRx significantly enhances microglial phagocytosis of myelin debris *in-vitro*. AstroRx transplantation *in-vivo* increased the percentage of microglia actively engulfing BODIPY^+^ myelin debris in the peri-lesion white matter. CBA analysis revealed that AstroRx helps maintain a balanced, nonpolarized microglial state, with decreased levels of IL-4, IL-6 and IL-17A, consistent with reparative activation without excessive inflammation, essential for CNS repair (Cherry et al., 2014). These effects were recapitulated in a human microglial model, where AstroRx enhanced phagocytic uptake of α-synuclein in a time-dependent manner. This supports the clinical translational potential of AstroRx, demonstrating preserved pro-reparative effects in a human microglial model mediated by both paracrine signaling and cell-cell interactions.

Second, immune homeostasis plays a vital role in CNS repair. AstroRx maintained lymphocyte proliferation and survival, without triggering excessive activation, a key characteristic of long-term immune regulation (Dombrowski et al., 2017). These immunomodulatory properties are vital for neuroprotection, allowing ongoing repair and preventing premature inflammation resolution, which limits impaired remyelination (Einstein et al., 2007).

Third, OPC differentiation and proliferation are key factors in efficient remyelination, supported by astrocyte-derived trophic signaling (Molina-Gonzalez et al., 2023). AstroRx enhanced OPC morphological maturation *in-vitro,* reflected by increased cellular extensions indicative of a functional differentiation state. Alongside increased differentiation into mature, morphologically complex O1^+^ oligodendrocytes, without affecting survival. These effects were validated *in-vivo*, where AstroRx transplantation led to an increase in NG2^+^ OPC and O1^+^ mature oligodendrocytes in the peri-lesion area, accompanied by a reduction in Ki67^+^ proliferating OPC and similar cell survival. To assess whether enhanced oligodendrogenesis translated into structural repair, myelin internode length was quantified in the peri-lesional white matter. AstroRx treatment showed a trend toward shorter internodes, consistent with newly formed myelin segments (Franklin and Ffrench-Constant, 2008; Young et al., 2013), supporting AstroRx role in promoting early-stage remyelination.

To further validate clinical translation, hESC-OPC exposed to AstroRx displayed reduced OPC proliferation, increased levels of O1^+^ oligodendrocytes and preserved cell survival, mirroring the effects observed in murine OPC.

One of the fundamental challenges in chronic demyelinating diseases, such as MS, is efficient drug delivery into the CNS, due to the compartmentalized nature of the disease and the partial closure of the blood-brain barrier (Lassmann, 2019). Intraventricular and subarachnoid delivery of pro-repair cell therapy offers a strategic advantage by positioning therapeutic cells in direct proximity to the demyelinated periventricular and spinal white matter tracts. This route optimizes the therapeutic bystander effect, providing neurotrophic support, while preserving the essential inflammatory processes required for effective repair. Our results suggest that intraventricularly delivered AstroRx acts through paracrine signaling to support local immune homeostasis and foster remyelination, without interfering with the inflammatory processes essential for regeneration. The therapeutic potential of this cell delivery utilizes AstroRx bystander effects more so by their superior effects in a transwell co-culture system over direct contact.

In conclusion, we show that AstroRx exhibits potent immunomodulatory and trophic properties, fostering a pro-repair inflammatory environment, enhancing myelin debris clearance, and promoting OPC differentiation into mature oligodendrocytes. By targeting the key biological barriers to remyelination, its scalability as an “off-the-shelf” cell product, and its safety following intra-thecal delivery in human clinical trials (Gotkine et al., 2023), AstroRx is positioned as a promising candidate for treating chronic demyelination, where multiple cellular dysfunctions must be addressed simultaneously.

## Resource availability

### Lead contact

Further information and requests for resources, reagents, and data for reanalysis should be directed to Tamir Ben-Hur (tamir@hadassah.org.il).

### Materials availability

This study did not generate new unique reagents.

### Data and code availability

This study did not generate datasets deposited in public repositories and does not report original code.

## Acknowledgments

This research was supported by a gift from the Judy & Sidney Swartz Foundation.

## Author contributions

Conceptualization, L.S., N.F., and T.B.H.; design, L.S., N.F., and T.B.H.; methodology, L.S., M.L., and T.G.; acquisition, L.S., M.L., T.G., Y.S., G.S., S.L., and D.S.; analysis, L.S.; interpretation of data, L.S., N.F., and T.B.H.; writing – original draft; L.S.; writing – review and editing: N.F., M.I., B.R., M.R., and T.B.H.

## Declaration of interests

G.S., S.L., and M.I. are employees of NewcelX Ltd.; M.R. is the CSO, director, and major shareholder; T.B.H. serves on its Scientific Advisory Board and received consulting fees and stock options. All other authors are not affiliated with NewcelX, declaring no competing interests.

## STAR★Methods

### Key resources table

**Table:** 

REAGENT or RESOURCE	SOURCE	IDENTIFIER
**Antibodies**		

anti-IBA1	Wako	Cat# 019–19741; RRID: AB_839504
anti-CD25	eBioscience	Cat# 35–025182; RRID: AB_11218898
anti-Bromo-deoxyuridine (BrdU) for Flow Cytometry	BD Biosciences	Cat# 347583; RRID: AB_400327
anti-CD3ε	eBioscience	Cat# 12-0031-82; RRID: AB_465496
anti-CD11c	eBioscience	Cat# 14-0114-82; RRID: AB_467115
anti-NG2	Millipore	Cat# AB5320; RRID: AB_91789
anti-BrdU for Histopathology	Bio-Rad	Cat# OBT0030G; RRID: AB_609568
anti-O1	R&D Systems	Cat# MAB1327; RRID: AB_357618
anti-Human Mitochondria	Biotest	Cat# MAB1273; RRID: AB_94052
anti-GFAP	Dako	Cat# Z0334; RRID: AB_10013382
anti-Ki67	Thermo Fisher Scientific	Cat# 14-5698-82; RRID: AB_10854564
anti-Neurofilament (NF)	Merck	Cat # MAB5448; RRID: AB_240862
goat anti-rabbit Alexa Fluor 555	Invitrogen	Cat# A-21428; RRID: AB_2535849
goat anti-rat Alexa Fluor 488	Invitrogen	Cat# A-11006; RRID: AB_2534074
goat anti-mouse Alexa Fluor 555	Invitrogen	Cat# A-21422; RRID: AB_141780
goat anti-rabbit Alexa Fluor 488	Invitrogen	Cat# A-11008; RRID: AB_143165
BODIPY	Invitrogen	Cat# D3922
DAPI	Vector Laboratories	Cat# H-1200; RRID: AB_2336790

**Bacterial and virus strains**		

Lipopolysaccharide (LPS)	Sigma-Aldrich	*Escherichia coli,* 111:B4

**Chemicals, peptides, and recombinant proteins**		

Percoll	Cytvia	Cat# GE17-0891-01
CD11b magnetic beads	Miltenyi Biotec	Cat #130-093-634
FITC-labeled α-synuclein	rPeptide	Cat# S-1113
Carboxyfluorescein succinimidyl ester (CFSE)	eBioscience	Cat# 65-0850
Low glucose DMEM	Sartorius	Cat# 01-050-1A
Fetal Bovine Serum	Gibco	Cat# 10270106
L-glutamine	Sartorius	Cat# 03-020-1C
Sodium pyruvate	Sartorius	Cat# 03-042-1B
Penicillin-streptomycin	Gibco	Cat# 15140-122
RPMI medium	Sartorius	Cat# 01-101-1A
MEM non-essential amino acids solution	Sartorius	Cat# 01-340-1B
β-mercapto-ethanol	Sigma-Aldrich	Cat# 63689
Concanavalin A (ConA)	Sigma-Aldrich	Cat# C5275
Gentamycin sulfate	Gibco	Cat# 15750-037
DMEM/F-12	Gibco	Cat# 31331-028
B27	Gibco	Cat# 12587-010
Basal Medium Eagle	Merck	B1522
IGF1	PeproTech	Cat# 100-11-100UG
3,3′,5-Triiodo-L-thyronine sodium salt (T3)	Sigma-Aldrich	Cat# T6397
Lysolecithin (LPC)	Sigma-Aldrich	Cat# L4129

**Critical commercial assays**		

Neural Tissue Dissociation Kit (P)	Miltenyi Biotec	Cat# 130-092-628
Th1/2/17 CBA kit	BD Biosciences	Cat# 560485; RRID: AB_2869354
Annexin V-FITC Apoptosis Kit	MBL Life Science	Cat# 4700
TUNEL *In Situ* Cell Death Detection Kit	Roche	Cat# 12156792910

**Experimental models: Cell lines**		

AstroRx®	NewcelX Ltd.	U.S. Patent No. 11406670 (Sonnenfeld et al., 2023)
HMC3 human microglial cells	ATCC	Cat# CRL-3304
Human ESC (HAD-C100)	Benjamin Reubinoff Laboratory	Culture: (Tannenbaum et al., 2012) Differentiation: (Nishri et al., 2020)

**Experimental models: Organisms/strains**		

Biozzi ABH mice	The Jackson Laboratory	https://www.jax.org/strain/027904
C57/BL6 mice	The Jackson Laboratory	https://www.jax.org/strain/000664

**Software and algorithms**		

ImageJ	National Institutes of Health	https://imagej.net/downloads RRID: SCR_003070
CBA software	BD	https://www.bdbiosciences.com/en-eu/products/reagents/immunoassays/cba
FCS express	De NoVo Software	https://denovosoftware.com/; RRID: SCR_016431
Prism	Graphpad	https://www.graphpad.com/features RRID: SCR_002798

**Other**		

MS columns	Miltenyi Biotec	Cat# 130-042-201
Transwell inserts	Getter Group	Cat# CA-3428
Hamilton syringe	Hamilton	Cat# 702SN

### Experimental model and study participant details

#### AstroRx

Clinical-grade cryopreserved off-the shelf AstroRx cell product, derived from hESC, was manufactured under current Good Manufacturing Practice (cGMP) conditions at NewcelX’s GMP facility. Production followed validated standard operating procedures ensuring consistency, sterility, and compliance with regulatory standards. Details on manufacturing processes and controls found in (Sonnenfeld et al., 2023). AstroRx is covered by U.S. Patent No. 11406670 held by Kadimastem Ltd (former company’s name).

#### Mice

All animal experiments were approved by the Hebrew University Institutional Animal Care and Use Committee (MD-19-16078-5 and MD-20-16227-1) and conducted in accordance with ethical regulations. Biozzi ABH mice and C57/BL6 mice (male and female) were bred and grown under specific pathogen-free conditions. For each experiment, mice age is indicated.

#### Primary microglia culture

Microglia were isolated from adult naive Biozzi ABH mice using the Neural Tissue Dissociation Kit (P) (Miltenyi Biotec, Cat# 130-092-628) as described (Lachish et al., 2022). Myelin debris were separate using Percoll (Cytvia, Cat# GE17-0891-01). CD11b^+^ cells were purified using magnetic beads (Miltenyi Biotec, Cat #130-093-634) and MS columns (Miltenyi Biotec, Cat# 130-042-201). Microglia were seeded on poly-D-lysine-coated coverslips (150,000 cells/mL) in low glucose DMEM (Sartorius, Cat# 01-050-1A) supplemented with 20% FBS (Gibco, Cat# 10270106), 20% L-cell conditioned medium, 1mM L-glutamine (Sartorius, Cat# 03-020-1C), sodium pyruvate (Sartorius, Cat# 03-042-1B), and penicillin-streptomycin (Gibco, Cat# 15140-122) at 37°C under 5% CO_2_.

#### Primary lymph node cell (LNC) culture

LNC were isolated from naive 8-12-week-old Biozzi ABH mice (Zveik et al., 2022). Cells were cultured (1 × 10^6^/mL) in RPMI medium (Sartorius, Cat# 01-101-1A) supplemented with 10% FBS, 1% MEM non-essential amino acids solution (Sartorius, Cat# 01-340-1B), 1mM sodium pyruvate, L-glutamine, penicillin-streptomycin, and β-mercapto-ethanol (Sigma-Aldrich, Cat# 63689) at 37°C under 5% CO_2_. LNC were co-cultured with AstroRx in direct contact (5:1 LNC: AstroRx ratio) or transwell (1:1 ratio) and stimulated with ConA (2.5 μg/mL; Sigma-Aldrich, Cat# C5275).

#### Primary OPC culture

Primary murine OPC were isolated from naive P0-P1 neonatal C57/BL6 mice cortices (Zveik et al., 2022). Mixed glial culture was grown in low-glucose DMEM with 5% FBS, 1mM sodium pyruvate, 1mM L-glutamine, and 0.6% gentamycin sulfate (Gibco, Cat# 15750-037) at 37°C under 5% CO_2_ for 9 days with media changes every 4 days. Microglia were removed by 30min shaking (140 rpm), then OPC were harvested by 18h shaking (200 rpm), using an orbital shaker. OPC were seeded (1 × 10^5^/mL) in DMEM/F-12 (Gibco, Cat# 31331-028) with 1% B27 supplement (Gibco, Cat# 12587-010) and 0.6% gentamycin sulfate on poly-D-lysine-coated coverslips. AstroRx co-culture was performed in direct contact (5:1) or transwell (1:1).

#### Human microglia cell line

HMC3 human microglial cells (ATCC, Cat# CRL-3304) were cultured in Basal Medium Eagle (Merck, Cat# B1522) with 10% FBS, penicillin-streptomycin and 1mM L-glutamine at 37°C under 5% CO_2_. AstroRx co-culture was performed in direct contact (5:1) or transwell (1:1).

#### hESC-derived OPC Culture

Human ESC (HAD-C100) were cultured as described (Tannenbaum et al., 2012). hESC-OPC were differentiated according to (Nishri et al., 2020) with minimal modifications at 37°C under 5% CO_2_. The OPC enriched culture was further differentiated on laminin for 10 days in SATO medium in the presence of IGF1 (10ng/mL) and T3 (60ng/mL). Cells were seeded and co-cultured under the same conditions as murine OPC.

### Method details

#### CFSE-labeled myelin debris

Myelin was harvested during microglial isolation and labeled with 2.5μM carboxyfluorescein succinimidyl ester (CFSE) (eBioscience, Cat# 65–0850) in a 2:1 (CFSE: myelin debris) ratio for 30min at room temperature. Excess CFSE was removed by four washes in 10% fetal bovine serum (FBS) in PBS (14,800 × g, 4°C, 10 min). Labeled myelin was suspended at 100 mg/mL and stored at −80°C (Rolfe et al., 2017).

#### Murine microglia phagocytosis assay

72 h (hr) after seeding, microglia were co-cultured with AstroRx in direct contact (5:1 ratio) or using transwell inserts (Getter Group, CA-3428) at a 1:1 ratio. LPS (200 ng/mL; Escherichia coli, 111:B4, Sigma-Aldrich) stimulation was applied 24h prior to adding CFSE-labeled myelin debris (5 μL/mL) at defined time points. Cells were immunostained with anti-Iba1 (1:220, Wako, Cat# 019–19741; RRID: AB_839504) and goat anti-rabbit Alexa Fluor 555 (1:200, Invitrogen, Cat# A-21428; RRID: AB_2535849); nuclei were counterstained with DAPI (Vector Laboratories, Cat# H-1200; RRID: AB_2336790). Confocal images were analyzed using ImageJ (NIH, RRID: SCR_003070) to quantify CFSE^+^ / Iba1^+^ cells and presented as mean ± SD from ≥3 independent experiments.

#### Cytokine quantification

Supernatant cytokine levels from murine microglia soups were analyzed using a mouse Th1/2/17 CBA kit (BD Biosciences, Cat# 560485; RRID: AB_2869354), as described (Ganz et al., 2023). Interleukin (IL)-2, IL-4, IL-6, IL-10, IL-17a, interferon-gamma (IFN-γ), and tumor necrosis factor-alpha (TNF-α) concentrations were measured by flow cytometry (BD LSRFortessa) and analyzed using CBA software.

#### Human microglia phagocytosis assay

HMC3 were incubated with FITC-labeled α-synuclein (2 mM, Cat# S-1113, rPeptide) for 60–300 min, then fixed and stained with anti-CD11c (eBioscience, Cat# 14-0114-82; RRID: AB_467115). Phagocytic uptake was quantified as described for murine microglia and analyzed using two-way ANOVA.

#### LNC flow cytometry assays

After 24h, LNC activation was assessed by CD25 expression (eBioscience, Cat# 35–025182; RRID: AB_11218898). Bromodeoxyuridine (BrdU, BD Biosciences, Cat# 347583; RRID: AB_400327) incorporation into CD3ε^+^ cells (eBioscience, Cat# 12-0031-82; RRID: AB_465496) and 2.5μM CFSE tracking measured proliferation after 72h. Calculation for the average number of cell divisions (Einstein et al., 2007):

$$\frac{\Sigma \;\frac{total\;events\;in\;cycle\;n}{2^{n}\;\left(for\;n\geq 1\right)}}{\Sigma \;\frac{total\;events\;in\;cycle\;n}{2^{n}\;\left(for\;n\geq 0\right)}}$$

Apoptosis was measured by Annexin V-FITC Apoptosis Kit (MBL Life Science, Cat# 4700). Data was collected using flow cytometry and analyzed with FCS express (De NoVo, RRID: SCR_016431), from ≥3 independent experiments.

#### *in-vitro* immunostaining assays

Cell surface staining was performed on living cells followed by fixation (Ganz et al., 2023), using anti-NG2 (1:200; Millipore, Cat# AB5320; RRID: AB_91789), anti-BrdU (1:200; Bio-Rad, Cat# OBT0030G; RRID: AB_609568) and anti-O1 (1:200; R&D Systems, Cat# MAB1327; RRID: AB_357618); secondary antibodies: goat anti-rabbit Alexa Fluor 555, goat anti-rat Alexa Fluor 488 (1:200; Invitrogen, Cat# A-11006; RRID: AB_2534074) and goat anti-mouse Alexa Fluor 555 (1:200; Invitrogen, Cat# A-21422; RRID: AB_141780). Cell apoptosis was evaluated using TUNEL *In Situ* Cell Death Detection Kit (Roche, Cat# 12156792910). Nuclei were stained by DAPI. Confocal images were analyzed using ImageJ and presented as mean ± SD from ≥3 independent experiments.

#### Oligodendrocyte morphological complexity analysis

FD analysis was performed using ImageJ, as described (Ganz et al., 2023). Individual cells were outlines and analyzed using the “Fractal box count” tool to obtain FD values. Skeleton analysis quantified branch length and endpoints using “Analyze Skeleton (2D/3D)”. ∼20 cells per condition per experiment were analyzed, totaling >150 cells.

#### Demyelination LPC model & cell transplantation

Biozzi ABH mice at 7 months of age, mice were anesthetized with ketamine (80 mg/kg; i.p.) and xylazine (20 mg/kg; i.p.) prepared in saline. A unilateral ICV transplantation of AstroRx (4 μL, 50,000 cells/μL), targeting stereotactic coordinates (bregma: 0mm; 1mm lateral; 2.2mm depth) with a Hamilton syringe (702SN, 25 μL) (Nishri et al., 2020) was performed; controls received saline. After 36h, mice were anesthetized again with the same protocol and injected unilaterally with 2 μL of 1% LPC (Sigma-Aldrich, Cat# L4129) at bregma: +1.2mm; 1.7mm lateral; 2.1mm depth. Mice were sacrificed on day seven. Groups consisted of six mice per condition.

#### Histopathology

Animals were anesthetized with a lethal dose of pentobarbital, and the brains were perfused via the ascending aorta with ice-cold PBS and 4% paraformaldehyde (Ben-Hur et al., 2003). Brains were deep-frozen, cryosectioned (10 μm coronal) and immunostained with anti-Iba1, anti-H.Mit (1:800; Biotest, Cat# MAB1273; RRID: AB_94052), anti-GFAP (1:200; Dako, Cat# Z0334; RRID: AB_10013382), anti-Ki67 (1:800; Thermo Fisher Scientific, Cat# 14-5698-82; RRID: AB_10854564), anti-NF (1:600; Merck, Cat # MAB5448; RRID: AB_240862), anti-NG2 and anti-O1. Secondary antibodies: goat anti-rabbit Alexa Fluor 555, goat anti-mouse Alexa Fluor 555, goat anti-rat Alexa Fluor 488, and goat anti-rabbit Alexa Fluor 488 (1:200; Invitrogen, Cat# A-11008; RRID: AB_143165). Nuclei were stained by DAPI. Microglial phagocytosis was assessed using anti-Iba1 and BODIPY (1:1000, Invitrogen, Cat# D3922), where sections were incubated with BODIPY for 15min, following secondary antibody incubation. Surface area of cells was measured using ImageJ. Myelin internode length was measured on continuous BODIPY^+^ segments aligned with NF^+^ axons, using ImageJ.

### Quantification and statistical analysis

Blind quantification was conducted independently by two researchers to ensure objectivity. Statistical analyses were performed using Prism (GraphPad; RRID: SCR_002798). Significance was determined by paired two-tailed Student’s *t* test and unpaired two-tailed Student’s *t-*test for two group comparisons, and one-way analysis of variance (ANOVA) or two-way ANOVA with Tukey’s multiple comparisons post hoc test for multiple comparisons. Significance level: ^∗^*p* < 0.05; ^∗∗^*p* < 0.01; ^∗∗∗^*p* < 0.001; ^∗∗∗∗^*p* < 0.0001, ns = not significant. Data are shown as mean ± standard deviation (SD). Sample size (n) are noted in figure legends.

## Supplemental information

Document S1. Figures S1–S3

Document S2. Article plus supplemental information

## References

[bib1] Aharonowiz M., Einstein O., Fainstein N., Lassmann H., Reubinoff B., Ben-Hur T. (2008). Neuroprotective Effect of Transplanted Human Embryonic Stem Cell-Derived Neural Precursors in an Animal Model of Multiple Sclerosis. PLoS One.

[bib2] Ben-Hur T. (2011). Cell Therapy for Multiple Sclerosis. Neurotherapeutics.

[bib3] Ben-Hur T., Einstein O., Mizrachi-Kol R., Ben-Menachem O., Reinhartz E., Karussis D., Abramsky O. (2003). Transplanted multipotential neural precursor cells migrate into the inflamed white matter in response to experimental autoimmune encephalomyelitis. Glia.

[bib4] Bourdette D., Wooliscroft L. (2024). Developing drugs that promote remyelination: Is our in vitro screening approach too simplistic?. Neurotherapeutics.

[bib5] Cherry J.D., Olschowka J.A., O’Banion M.K. (2014). Neuroinflammation and M2 microglia: The good, the bad, and the inflamed. J. Neuroinflammation.

[bib6] Dombrowski Y., O’Hagan T., Dittmer M., Penalva R., Mayoral S.R., Bankhead P., Fleville S., Eleftheriadis G., Zhao C., Naughton M. (2017). Regulatory T cells promote myelin regeneration in the central nervous system. Nat. Neurosci..

[bib7] Einstein O., Fainstein N., Vaknin I., Mizrachi-Kol R., Reihartz E., Grigoriadis N., Lavon I., Baniyash M., Lassmann H., Ben-Hur T. (2007). Neural precursors attenuate autoimmune encephalomyelitis by peripheral immunosuppression. Ann. Neurol..

[bib8] Franklin R.J.M., Ffrench-Constant C. (2008). Remyelination in the CNS: From biology to therapy. Nat. Rev. Neurosci..

[bib9] Franklin R.J.M., Simons M. (2022). CNS remyelination and inflammation: From basic mechanisms to therapeutic opportunities. Neuron.

[bib10] Galipeau J., Sensébé L. (2018). Mesenchymal Stromal Cells: Clinical Challenges and Therapeutic Opportunities. Cell Stem Cell.

[bib11] Ganz T., Zveik O., Fainstein N., Lachish M., Rechtman A., Sofer L., Brill L., Ben-Hur T., Vaknin-Dembinsky A. (2023). Oligodendrocyte progenitor cells differentiation induction with MAPK/ERK inhibitor fails to support repair processes in the chronically demyelinated CNS. Glia.

[bib13] Gotkine M., Caraco Y., Lerner Y., Blotnick S., Wanounou M., Slutsky S.G., Chebath J., Kuperstein G., Estrin E., Ben-Hur T. (2023). Safety and efficacy of first-in-man intrathecal injection of human astrocytes (AstroRx®) in ALS patients: Phase I/IIa clinical trial results. J. Transl. Med..

[bib14] Hastings N., Kuan W.-L., Osborne A., Kotter M.R.N. (2022). Therapeutic Potential of Astrocyte Transplantation. Cell Transplant..

[bib15] Izrael M., Slutsky S.G., Admoni T., Cohen L., Granit A., Hasson A., Itskovitz-Eldor J., Krush Paker L., Kuperstein G., Lavon N. (2018). Safety and efficacy of human embryonic stem cell-derived astrocytes following intrathecal transplantation in SOD1G93A and NSG animal models. Stem Cell Res. Ther..

[bib16] Kotter M.R., Li W.-W., Zhao C., Franklin R.J.M. (2006). Myelin Impairs CNS Remyelination by Inhibiting Oligodendrocyte Precursor Cell Differentiation. J. Neurosci..

[bib17] Lachish M., Fainstein N., Ganz T., Sofer L., Ben-Hur T. (2022). Failure of Alzheimer’s Mice Brain Resident Neural Precursor Cells in Supporting Microglia-Mediated Amyloid β Clearance. Cells.

[bib18] Lassmann H. (2018). Pathogenic Mechanisms Associated With Different Clinical Courses of Multiple Sclerosis. Front. Immunol..

[bib19] Lassmann H., Bradl M. (2017). Multiple sclerosis: Experimental models and reality. Acta Neuropathol..

[bib20] Lloyd A.F., Miron V.E. (2019). The pro-remyelination properties of microglia in the central nervous system. Nat. Rev. Neurol..

[bib21] Miron V.E., Boyd A., Zhao J.-W., Yuen T.J., Ruckh J.M., Shadrach J.L., van Wijngaarden P., Wagers A.J., Williams A., Franklin R.J.M., ffrench-Constant C. (2013). M2 microglia and macrophages drive oligodendrocyte differentiation during CNS remyelination. Nat. Neurosci..

[bib22] Molina-Gonzalez I., Holloway R.K., Jiwaji Z., Dando O., Kent S.A., Emelianova K., Lloyd A.F., Forbes L.H., Mahmood A., Skripuletz T. (2023). Astrocyte-oligodendrocyte interaction regulates central nervous system regeneration. Nat. Commun..

[bib23] Nishri Y., Hampton D., Ben-Shushan E., Fainstein N., Magnani D., Aharonowiz M., Reubinoff B.E., Chandran S., Ben-Hur T. (2020). Continuous Immune-Modulatory Effects of Human Olig2+ Precursor Cells Attenuating a Chronic-Active Model of Multiple Sclerosis. Mol. Neurobiol..

[bib24] Nutma E., van Gent D., Amor S., Peferoen L.A.N. (2020). Astrocyte and Oligodendrocyte Cross-Talk in the Central Nervous System. Cells.

[bib25] Plemel J.R., Michaels N.J., Weishaupt N., Caprariello A.V., Keough M.B., Rogers J.A., Yukseloglu A., Lim J., Patel V.V., Rawji K.S. (2018). Mechanisms of lysophosphatidylcholine-induced demyelination: A primary lipid disrupting myelinopathy. Glia.

[bib26] Rolfe A.J., Bosco D.B., Broussard E.N., Ren Y. (2017). In Vitro Phagocytosis of Myelin Debris by Bone Marrow-Derived Macrophages. J. Vis. Exp..

[bib27] Smith J.A., Nicaise A.M., Ionescu R.-B., Hamel R., Peruzzotti-Jametti L., Pluchino S. (2021). Stem Cell Therapies for Progressive Multiple Sclerosis. Front. Cell Dev. Biol..

[bib28] Sonnenfeld T., Rauchbach E., Downey R., Blumenkrants D., Hasson A., Kuperstein G., Kronfeld N., Margalit R., Molak K., Morad V. (2023). Toxicity Studies on Intrathecal Injection of Low Dose of DMSO Used for Cryopreservation of Human Astrocytes in Mice. J. Clin. Toxicol..

[bib29] Tannenbaum S.E., Turetsky T.T., Singer O., Aizenman E., Kirshberg S., Ilouz N., Gil Y., Berman-Zaken Y., Perlman T.S., Geva N. (2012). Derivation of Xeno-Free and GMP-Grade Human Embryonic Stem Cells – Platforms for Future Clinical Applications. PLoS One.

[bib30] Toth E., Rassul S.M., Berry M., Fulton D. (2021). A morphological analysis of activity-dependent myelination and myelin injury in transitional oligodendrocytes. Sci. Rep..

[bib31] Uccelli A., Laroni A., Freedman M.S. (2013). Mesenchymal stem cells as treatment for MS - progress to date. Mult. Scler..

[bib32] Young K.M., Psachoulia K., Tripathi R.B., Dunn S.-J., Cossell L., Attwell D., Tohyama K., Richardson W.D. (2013). Oligodendrocyte Dynamics in the Healthy Adult CNS: Evidence for Myelin Remodeling. Neuron.

[bib33] Zveik O., Fainstein N., Rechtman A., Haham N., Ganz T., Lavon I., Brill L., Vaknin-Dembinsky A. (2022). Cerebrospinal fluid of progressive multiple sclerosis patients reduces differentiation and immune functions of oligodendrocyte progenitor cells. Glia.

