# Supplementary material for: Pro-repair properties of a human embryonic stem cell-derived astrocyte cell therapy in demyelinating disorders
Source: Stem Cell Reports. 2026 Jun 11;21(7):102960. doi: 10.1016/j.stemcr.2026.102960 (PMC13385419; doi:10.1016/j.stemcr.2026.102960)
Supplement: Document S1. Figures S1–S3 [file mmc1.pdf]

Stem Cell Reports, Volume *21*

# Supplemental Information

## Pro-repair properties of a human embryonic stem cell-derived astro-cyte cell therapy in demyelinating disorders

**Lihi Sofer Stepanov, Nina Fainstein, Marva Lachish, Tal Ganz, Yoel Shor, Gal Lebiush Shalom, Shir Klein-Lavi, Michal Izrael, Debora Steiner, Benjamin E. Reubinoff, Michel Revel, and Tamir Ben-Hur**

**Pro-repair properties of a human embryonic stem cell – derived astrocyte cell therapy in demyelinating disorders**

**Supplemental Information:**

**Supplemental Figures S1 – S3 with legends**

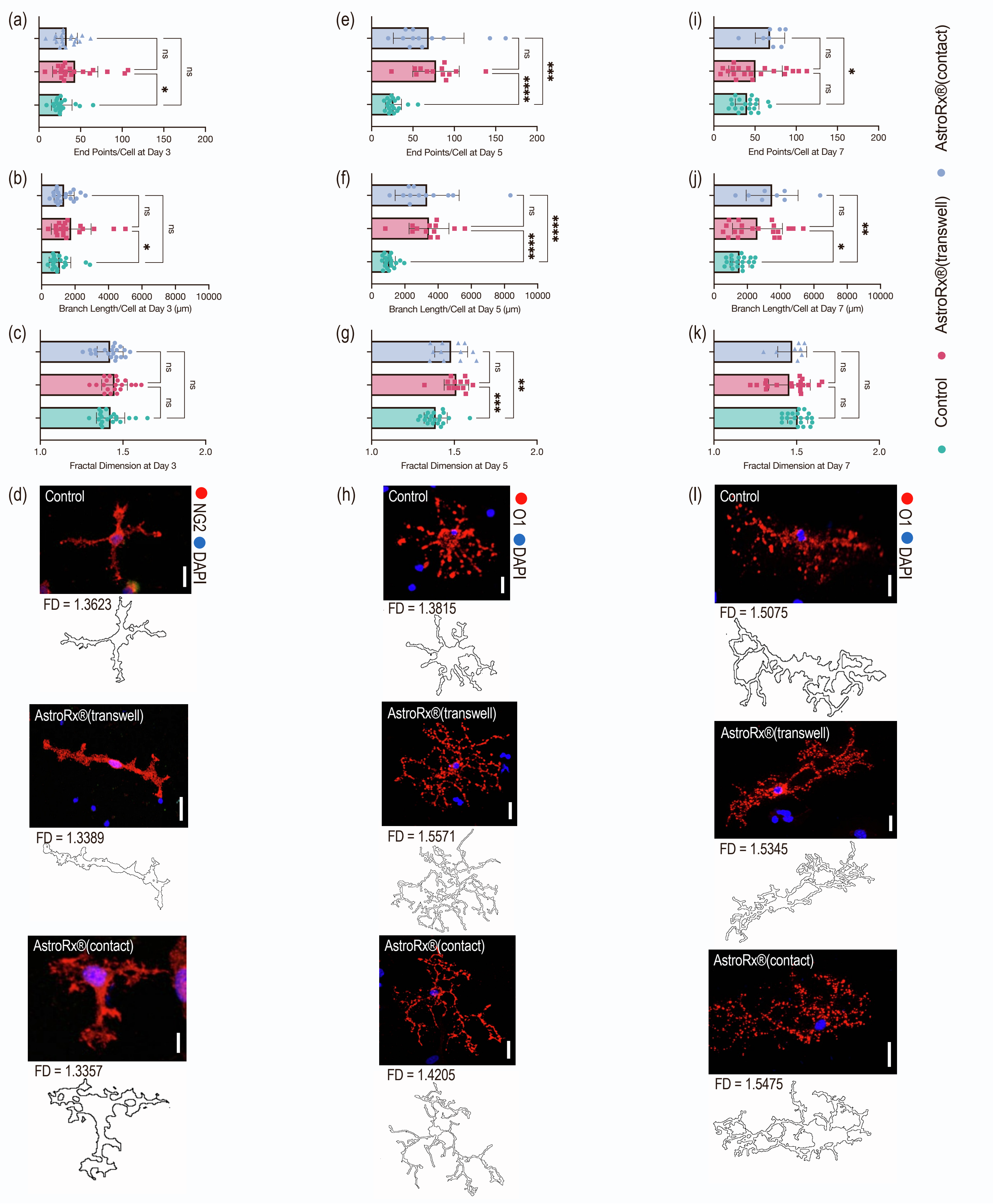
(a)
End Points/Cell at Day 3
(b)
Branch Length/Cell at Day 3 (μm)
(c)
Fractal Dimension at Day 3
(d)
Control
NG2
DAPI
FD = 1.3623
AstroRx®(transwell)
FD = 1.3389
AstroRx®(contact)
FD = 1.3357
(e)
End Points/Cell at Day 5
(f)
Branch Length/Cell at Day 5 (μm)
(g)
Fractal Dimension at Day 5
(h)
Control
O1
DAPI
FD = 1.3815
AstroRx®(transwell)
FD = 1.5571
AstroRx®(contact)
FD = 1.4205
(i)
End Points/Cell at Day 7
(j)
Branch Length/Cell at Day 7 (μm)
(k)
Fractal Dimension at Day 7
(l)
Control
O1
DAPI
FD = 1.5075
AstroRx®(transwell)
FD = 1.5345
AstroRx®(contact)
FD = 1.5475
AstroRx®(contact)
AstroRx®(transwell)
Control

**Figure S1: AstroRx® promote murine Oligodendrocyte Morphological Complexity by Fractal Dimension & Skeleton Analysis *in-vitro.***

(a) Skeleton analysis showed increased endpoints/cell at day 3 with AstroRx®; control (27.35 ± 12.42), AstroRx®(transwell) (43.55 ± 27.16), AstroRx®(contact) (33.25 ± 12.93), $p = 0.028$.
(b) Branch length/cell was higher at day 3 in AstroRx®-treated cultures; control (1100 ± 649 µm), AstroRx®(transwell) (1771 ± 1188 µm), AstroRx®(contact) (1346 ± 600 µm), $p = 0.051$.
(c) FD analysis revealed no differences in morphological complexity on day 3; control (1.43 ± 0.06), AstroRx®(transwell) (1.45 ± 0.08), AstroRx®(contact) (1.42 ± 0.08), $p = 0.521$.
(d) Representative images of NG2+ OPC on day 3 and cell outline with FD values.
(e-f) AstroRx® increased endpoints and branch length at day 5 (e) endpoints/cell: control (26.45 ± 9.65), AstroRx®(transwell) (77.92 ± 28.07), AstroRx®(contact) (69.00 ± 42.71), $p < 0.001$). (f) branch length/cell: control (1075 ± 351 µm), AstroRx®(transwell) (3452 ± 1198 µm), AstroRx®(contact) (3341 ± 1927 µm), $p < 0.001$.
(g) FD analysis exhibited significantly increased complexity at day 5; control (1.39 ± 0.07), AstroRx®(transwell) (1.51 ± 0.07), AstroRx®(contact) (1.48 ± 0.10), $p < 0.001$.
(h) Representative images of O1+ oligodendrocytes at days 5 and cell outline with FD values.
(i-j) AstroRx® increased endpoints and branch length at day 7 (i) endpoints/cell: control (40.40 ± 14.30), AstroRx®(transwell) (50.65 ± 32.44), AstroRx®(contact) (68.25 ± 17.83), $p = 0.028$. (j) branch length/cell: (control (1535 ± 569 µm), AstroRx®(transwell) (2607 ± 1501 µm), AstroRx®(contact) (3490 ± 1557 µm), $p = 0.0007$.
(k) No difference in FD score on day 7 was observed; control (1.51 ± 0.061), AstroRx®(transwell) (1.46 ± 0.13), AstroRx®(contact) (1.47 ± 0.09), $p = 0.275$;.
(l) Representative images of O1+ oligodendrocytes at days 7 and cell outline with FD values.
Scale bar = 30 µm. n = 3 exp. Significance was determined by one-way ANOVA with Tukey's post hoc test.

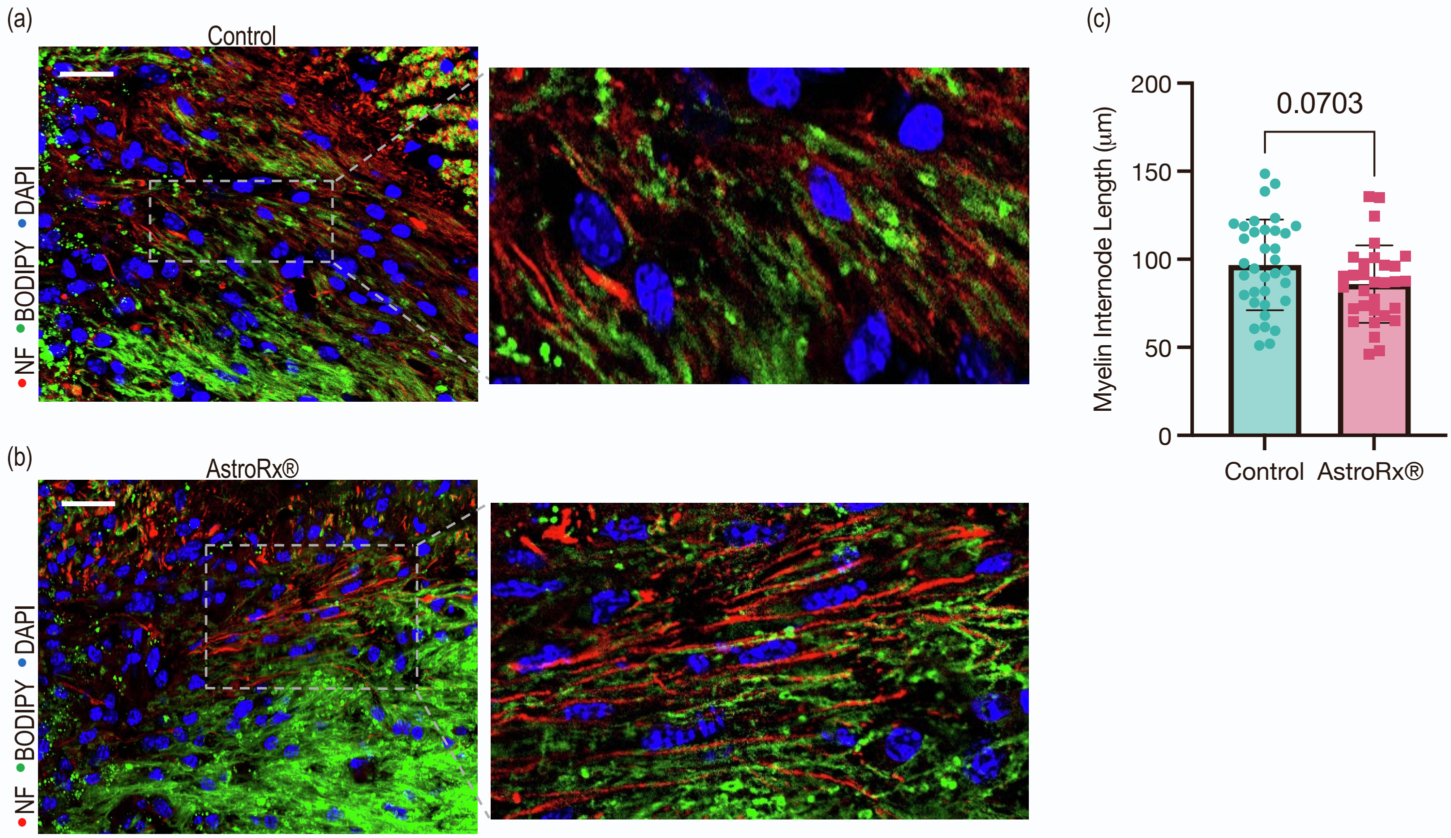
(a)
Control
NF BODIPY DAPI
(b)
AstroRx®
NF BODIPY DAPI
(c)
0.0703
Myelin Internode Length (μm)
200
150
100
50
0
Control
AstroRx®

**Figure S2. Myelin internode length analysis in peri-lesional white matter following AstroRx® transplantation.**
(a-b) Immunofluorescence analysis of BODIPY+ NF+ myelinated internodes. Dashed box indicates region shown at higher magnification of flattened confocal z-stack images.
(c) Quantification of myelin internode length (µm) measured along NF+ axons.
Scale bar = 50 µm; n = 6 mice; ≥35 internodes per mouse. Significance was determined by student's unpaired t-test.

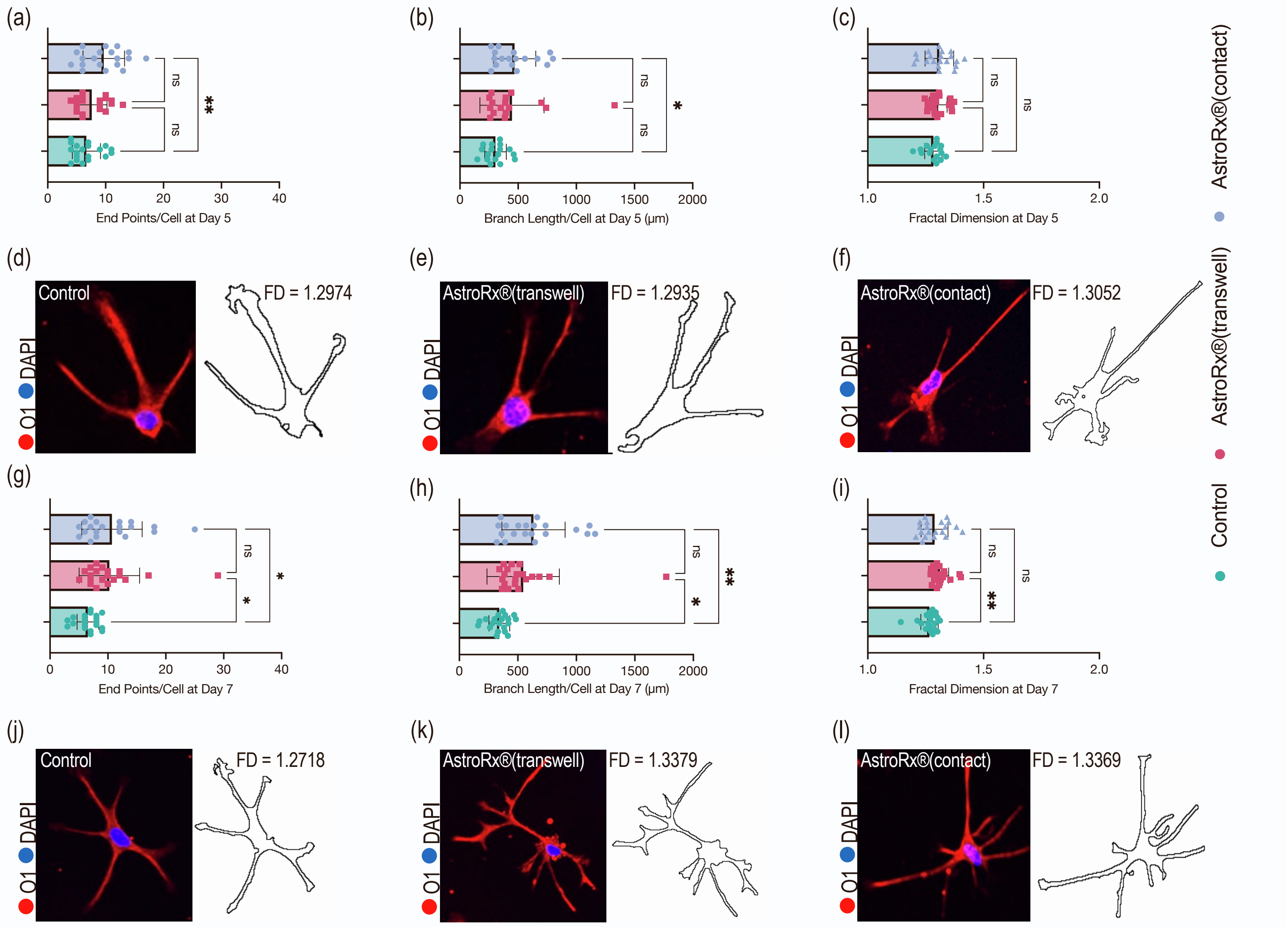
(a)
End Points/Cell at Day 5
ns
ns
**
(b)
Branch Length/Cell at Day 5 (μm)
ns
ns
*
(c)
Fractal Dimension at Day 5
ns
ns
ns
AstroRx®(contact)
AstroRx®(transwell)
Control
(d)
Control
FD = 1.2974
O1 DAPI
(e)
AstroRx®(transwell)
FD = 1.2935
O1 DAPI
(f)
AstroRx®(contact)
FD = 1.3052
O1 DAPI
(g)
End Points/Cell at Day 7
ns
*
*
(h)
Branch Length/Cell at Day 7 (μm)
ns
*
**
(i)
Fractal Dimension at Day 7
ns
**
ns
(j)
Control
FD = 1.2718
O1 DAPI
(k)
AstroRx®(transwell)
FD = 1.3379
O1 DAPI
(l)
AstroRx®(contact)
FD = 1.3369
O1 DAPI

**Figure S3: AstroRx® enhance Morphological Complexity hESC-derived Oligodendrocytes *in-vitro.***
(a-b) Skeleton analysis shows higher endpoints and branch length per cell at day 5 in AstroRx®-treated cultures (a) endpoints/cell: control (6.68 ± 2.43), AstroRx®(transwell) (7.62 ± 2.62), AstroRx®(contact) (9.68 ± 3.59), p = 0.008. (b) branch length/cell: control (305.5 ± 92.34 µm), AstroRx®(transwell) (447.1 ± 275.7 µm), AstroRx®(contact) (469.8 ± 181.4 µm), p = 0.039.
(c) FD analysis revealed no differences in morphological complexity on day 5; control (1.28 ± 0.04), AstroRx®(transwell) (1.30 ± 0.04), AstroRx® (contact)(1.31 ± 0.06), p = 0.243.
(d-f) Representative images of O1+ oligodendrocytes at day 5 and cell outline with FD values.
(g-h) AstroRx® increased endpoints and branch length at day 7 (g) endpoints/cell: control (6.55 ± 1.88), AstroRx®(transwell) (10.25 ± 5.22), AstroRx®(contact) (10.70 ± 5.22), *p* = 0.007. (h) branch length/cell: control (341.6 ± 88.41 µm), AstroRx®(transwell) (546.0 ± 309.5 µm), AstroRx®(contact) (633.5 ± 270.0 µm), p = 0.001.
(i) FD analysis exhibited significantly increased complexity at day 7; control (1.27 ± 0.04), AstroRx®(transwell) (1.31 ± 0.04), AstroRx®(contact) (1.29 ± 0.06), *p* = 0.008.
(j-l) Representative images of O1+ oligodendrocytes at day 7 and cell outline with FD values.
Scale bar = 30 µm. n = 3 exp. Significance was determined by one-way ANOVA with Tukey's post hoc test.
